# The suitability and attraction of the population of the United Arab Emirates for the study of genetic inheritance in scoliosis

**DOI:** 10.1186/1748-7161-7-S1-O72

**Published:** 2012-01-27

**Authors:** K Bagnall, A Al Kaabi, H Al Hamsi, T Al Mazrouee, A D’souza

**Affiliations:** 1 UAE University, Al Ain, United Arab Emirates

## Background

Recently, significant advances have been made in understanding the aetiology of adolescent idiopathic scoliosis (AIS) in the area of genetic inheritance. Essential to the success of these studies is the finding of large families with several cases of AIS from which blood samples can be collected and analysed. The emirati culture is unique and encourages large families with many children, often several wives with the same husband, and many cases of consanguinity especially among first cousins. This complex family structure might very well be suited to provide more appropriate examples for the study of genetic heritage in AIS than the more typical western family.

## Material and methods

The genealogical history of 15 extended emirati families was collected for 4 generations which would approximate that required for the study of AIS. The number of children in each family and the incidence of consanguinity among first cousins were calculated. These data were compared with similar, published data for western families.

## Results

The average number of children per family among the emirati population was 6.29 (often >10), approximately 3x that of the average western family. In each family there were several examples of multiple wives and first cousin marriages. Two families were found in which at least one person had AIS.

## Conclusions

These results suggest that the unique, well controlled structure of the emirati families lend themselves to being excellent for extensive study of the genetic inheritance aspects of the aetiology of AIS because of the greater familial component.

